# Involving community pharmacies in management of late effects of cancer treatment: Opinions from cancer survivors.

**DOI:** 10.1016/j.rcsop.2024.100514

**Published:** 2024-09-21

**Authors:** Nadia Lund Olsen, Ramune Jacobsen, Linda Aagaard Thomsen, Lotte Stig Nørgaard

**Affiliations:** a Health care professional consultant, Cancer Survivorship, Danish Cancer Institute, The Danish Cancer Society, Denmark; b Social and Clinical Pharmacy, Department of Pharmacy, University of Copenhagen, Denmark; c Head of Department, Department for Rare Diseases, The Danish Medicines Council, Denmark

**Keywords:** Community pharmacy, cancer survivor, Pharmacist, Late effects of cancer, Survivorship

## Abstract

**Background:**

More than 50 % of cancer survivors experience late effects of cancer (LEC). Current models of follow-up care often prove inadequate, resulting in unresolved LEC. Given the pivotal role of community pharmacists as the most accessible healthcare professionals and the demonstrated benefits of evidence-based pharmacy services on patient centered care, exploring the potential contribution of community pharmacies in managing LEC is relevant.

**Objective:**

This study aimed to investigate cancer survivors' needs, preferences, and attitudes regarding pharmacy involvement in managing LEC.

**Method:**

The developed questionnaire based on validated instruments underwent a pilot test among cancer survivors at four Danish community pharmacies. In August 2021, the questionnaire was distributed to all 611 cancer survivors of the Danish Cancer Society's cancer patient panel. The resulting quantitative data were subjected to descriptive statistical analysis, while qualitative data underwent a thematic content analysis.

**Results:**

Among the 611 panel members, 354 responded to the questionnaire (response rate 58 %). Fatigue was the most frequent LEC experienced by 88 % of respondents. Three out of four (75 %) of respondents expressed dissatisfaction with the level of counseling they had received regarding LEC, and 23 % disclosed not having discussed LEC with a healthcare professional despite feeling the need to do so. Nearly all respondents visited pharmacies annually and used available products to alleviate LEC. Approximately half of respondents expressed a need for counseling on the appropriate use of these products. While nearly half of respondents were receptive to pharmacy-based counseling, concerns regarding discretion and staff knowledge were prevalent.

**Conclusion:**

Cancer survivors experience an insufficiency in counseling on LEC and demonstrate an openness towards involving community pharmacies in addressing this gap. However, further investigation is warranted to delineate survivors' specific needs and expectations regarding community pharmacy involvement in LEC management. Additionally, suggestions from survivors underscore the importance of enhancing pharmacy staff knowledge and establishing discreet counseling areas.

## Highlights

• Cancer survivors express the need for better guidance on LEC management.
• Nearly half (48 %) of respondents support pharmacy-based counseling for LEC management.
• Half of the respondents visited a community pharmacy weekly or monthly.
• More than a third (39 %) lacked awareness of how pharmacies could assist with LEC.
• Improved privacy and staff knowledge are needed for effective pharmacy counseling.

## 1 Introduction

Since cancer incidence and survival rates continually increase, the population of cancer survivors is also rising.^1^ Over 50 % of cancer survivors experience one or more late effects of cancer (LEC) following the disease and treatment.^2^ LEC encompasses health issues that may arise during primary cancer treatment, evolving into chronic conditions or manifesting months or even years after treatment cessation, such as fatigue, digestive problems, and anxiety.^3^ In this study, cancer survivors were defined as individuals living with or after cancer, including both current and former cancer patients.

Effective prevention and management of LEC are crucial for enhancing cancer survivors' health-related quality of life, overall health status, and work ability.^4^ From the standpoint of the healthcare system, managing LEC presents challenges due to the shared responsibility for treatment and rehabilitation among hospitals, general practitioners (GPs), and municipalities.^5^ This is further complicated by the fact that LEC may develop over time.^4^ Existing models of follow-up care have proven inadequate in addressing the needs of many cancer survivors, resulting in unresolved LEC.^5^^,^^6^

Community pharmacists represent the most accessible healthcare professionals (HCPs) for the public.^7^^,^^8^ Denmark, with a population of 6 million, hosts more than 500 community pharmacies, serving over 3 million visitors monthly and maintaining contact with 94 % of the Danish adult population annually.^9^ The highly educated workforce at community pharmacies with both pharmacists and pharmacy technicians offers an extensive range of public health services.^10^ Since the early 2000s, Danish community pharmacies have increasingly expanded their role within the healthcare system. Evidence-based pharmacy counseling services and patient-centered interventions have demonstrated improvements in physical health status, health-related quality of life, patient self-care, treatment adherence, knowledge, satisfaction, and lower mortality rates.^11^, ^12^, ^13^, ^14^, ^15^ This development has positioned community pharmacies as key players in the interdisciplinary management of chronic conditions, further supported by consistent evidence based evaluations and adaptations.

Previous research from other countries has successfully engaged pharmacists in cancer awareness and screening,^16^, ^17^, ^18^, ^19^, ^20^, ^21^, ^22^, ^23^, ^24^, ^25^, ^26^ counseling on treatment adherence and side effects,^27^, ^28^, ^29^, ^30^ cancer prevention efforts, including smoking cessation and nutrition advice, as well as administering HPV vaccines^31^^,^^32^ and providing lifestyle interventions for prostate cancer survivors.^33^^,^^34^ Despite these advancements, there are currently no community pharmacy-based services specifically tailored to managing LEC in Denmark. Moreover, studies investigating the potential role of community pharmacists in LEC management within the Danish context are lacking. Thus, it is relevant to explore how Danish community pharmacies might facilitate interdisciplinary collaboration and contribute to the management of LEC and assess the interest of cancer survivors in utilizing such services.

This study aimed to explore cancer survivors' needs, preferences, and experiences regarding counseling on LEC and their attitudes towards involving community pharmacies in LEC management. The findings will inform future development of a community-pharmacy based intervention.

## 2 Method

### 2.1 Data collection and setting

The study utilized data gathered through a questionnaire targeting cancer survivors in Denmark. A questionnaire was distributed among members of the Danish Cancer Society (DCS) User Panel.^35^ The panel includes both current and former cancer patients (cancer survivors), as well as their relatives and next-of-kin. The panelists are volunteers, and the panel is engaged in various research activities, polls, case-studies, and discussions on political matters facilitated by the DCS. The DCS has obtained written consent from all User Panel members, and data collected within the panel adheres to the guidelines outlined in the General Data Protection Regulation (GDPR). For the purposes of this study, only cancer survivors were included. At the time of distribution, the panel consisted of 611 cancer survivors. Respondents ranged from 18 to 80+ years old, with 69 % of them being women. The year of cancer diagnosis spanned from January 2010 to December 2020.

### 2.2 Questionnaire

Developed collaboratively by researchers from the DCS and the University of Copenhagen, the questionnaire was constructed by adapting previously validated instruments, such as the 2019 Barometer survey by the DCS^6^^,^^36^, ^37^, ^38^ with modifications made to suit the study objectives.

Comprising 19 questions (see supplementary material), the questionnaire was organized into five thematic sections: respondent characteristics (Q1, Q2, Q19), experienced LEC and general experiences with management of LEC (Q3-Q8), general experience with community pharmacy counseling (Q9-Q11), use of pharmacy products for LEC alleviation (Q12-Q15), and perceptions and utilization of community pharmacies in LEC management (Q16-Q18). The response options were either dichotomous or used categorical frequency, extent, or agreement rating scales. Certain questions (Q4, Q6, Q10, Q12, Q13) allowed for free-text elaboration.

The questionnaire underwent a pilot test and subsequent adaptation in collaboration with the Department of Patient Support and Voluntary Activities at DCS, responsible for the User Panel, to ensure face and content validity. Conducted by seven community pharmacy interns across four pharmacies in the spring 2021, the pilot involved the recruitment of 18 respondents through promotional materials placed within the pharmacies. Respondents completed the pilot questionnaire via Google Forms on tablets and computers in the pharmacies. The pilot affirmed the face validity of the questions, with minor linguistic adjustments made, such as translating professional terminology into layman terms. The questionnaire was then set up in SurveyXact (an online survey software developed by Ramboll, Denmark^39^).

### 2.3 Data collection

On August 16th, 2021, the questionnaire was distributed via email to the 611 cancer survivors comprising the DCS User Panel. Respondents were given a two-week window to complete the questionnaire, with a reminder sent after one week to enhance the response rate. SurveyXact provided the access to the questionnaire.

### 2.4 Statistics and data analysis

Quantitative data were analyzed using descriptive statistics in Microsoft Excel®, with frequencies calculated for each response category to summarize response distributions.

Analysis of free-text responses employed a thematic content analysis with an inductive approach, inspired by Braun & Clarke.^40^ Key themes were identified through familiarization with the data, coding, theme identification, and review processes, with ten themes established and illustrated using quotes.^41^ The analysis was conducted both manually and utilizing Microsoft Excel. All quotes were translated from Danish to English by the authors.

Quantitative and qualitative results are presented collectively under key themes delineated as section headings in the results section.

## 3 Results

### 3.1 Respondents

In total, 354 of the 611 members of the User Panel initiated the questionnaire, resulting in a response rate of 58 %. Among these respondents, 30 did not complete the questionnaire; however, their partially completed responses were deemed substantial enough to be included in the data analysis. The survey yielded 700 free-text entries.

Of the 354 respondents, 47 % reported that they are currently cancer-free and no longer undergo treatment, while 50 % reported receiving treatment. The remaining 3 % stated that they had never undergone cancer treatment. A total of 53 % of respondents indicated that their treatment had concluded between 2 and 20 years ago, as presented in Table 1. Additional demographic data, including sex, age, and education, as well as the diagnoses of the respondents, are presented in Table 1.

**Table 1:** – Demographic data for the respondents completing the questionnaire.

	**Number**	**Percentage**
**Respondents**		
Female	227	70.1 %
Male	97	299 %

**Age group**		
18–29 years	1	0.3 %
30–39 years	12	3.7 %
40–49 years	37	11.4 %
50–59 years	78	24.1 %
60–69 years	124	38.3 %
70–79 years	67	20.7 %
80+ years	5	1.5 %

**Educational level**		
Primary and lower secondary school	14	4.3 %
Vocational education	63	19.5 %
Upper secondary school	20	6.2 %
Short-cycle higher education (1–2 years)	44	13.6 %
Medium-cycle higher education (3–4 years)	129	39.9 %
Long-cycle higher education (≥5 years)	52	16.1 %
Other	1	0.3 %

**Occupation**		
Enrolled in education (e.g. student, apprentice)	4	1.2 %
In paid employment	76	23.5 %
Self-employed	10	3.1 %
Unemployed or job seeking	6	1.9 %
On sick leave	17	5.3 %
Subsidized employment (due to substantial impaired work capacity)	21	6.5 %
On leave (maternity, nursing or other forms of leave)	0	0.0 %
Retired and early retirement	267	53.2 %
Other	17	5.3 %

**Cancer type**		
Head and neck cancer	23	7.1 %
Breast cancer	106	32.7 %
Gastric−/bowel cancer	43	13.3 %
Pancreatic cancer	5	1.5 %
Lung cancer	11	3.4 %
Urethral cancer	8	2.5 %
Pelvic cancer, women	21	6.5 %
Pelvic cancer, men	2	0.6 %
Bladder cancer	31	9.6 %
Blood cancer and lymphoma	32	9.9 %
Sarcomas	4	1.2 %
Brain and nervous system cancer	3	0.9 %
Malignant melanoma	12	3.7 %
Other skin cancer than melanoma	3	0.9 %
Diagnose unknown	1	0.3 %
Other cancer diseases	19	5.9 %

**Time since cancer treatment finished**		
The treatment is not finished	110	32.0 %
0–1 years	52	15.1 %
2–5 years	109	31.7 %
6–10 years	59	17.2 %
11–15 years	10	2.9 %
16–20 years	3	0.9 %
21 years or longer	1	0.3 %

### 3.2 Cancer survivors' LEC and experiences with management of LEC

**Fig. 1:**
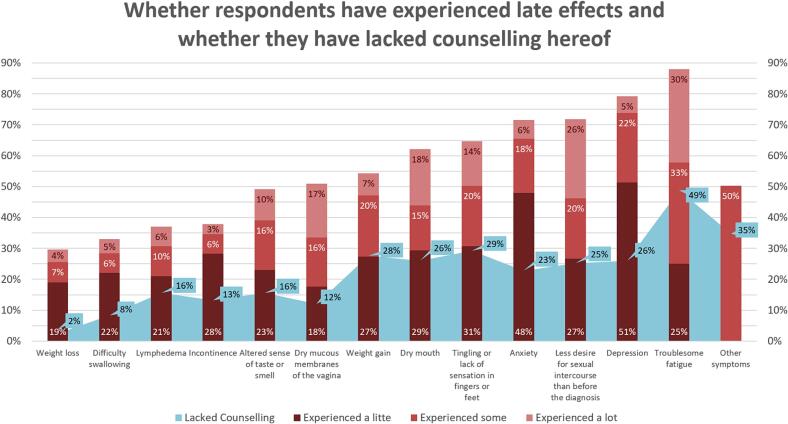
The stacked columns illustrate the extent to which cancer survivors have encountered various LEC, while the area curve indicates whether they have received adequate counseling for each specific LEC.

In Fig. 1, the bars depict the prevalence of different types of LEC experienced by respondents, and the area curve illustrates the magnitude of inadequate counseling across various types of LEC. The most frequently reported LEC among respondents was fatigue, reported by 88 % of respondents. Additionally, 50 % of respondents reported experiencing other LECs not initially listed in the questionnaire, with further details provided in free-text responses. These responses primarily highlighted physical LEC, such as hormonal changes, pain, and functional impairments, as well as psychological LEC including mental health problems and personality alterations. Furthermore, ten responses highlighted social functioning challenges stemming from fatigue, cognitive changes, and gastrointestinal issues.

A substantial majority (75 %) of respondents expressed dissatisfaction with the level of counseling they had received regarding LEC.

In the free-text responses, numerous respondents highlighted a general lack of information regarding both treatment side effects and LEC, expressing surprise at encountering these phenomena both during and after treatment. Many expressed a need for additional guidance on managing LEC in their daily lives, with a particular emphasis on counseling for pain management. Additionally, some respondents indicated a desire for information on distinguishing symptoms of potential relapse from those of LEC, as exemplified by the following quote:

“As a cancer patient, you live in constant fear of the cancer returning. Therefore, information about LEC is important because they may be mistaken as signs of illness.”

One-third of respondents (32 %) reported having had a constructive dialogue with a Health Care Professional (HCP) regarding their LEC. Another third (33 %) indicated having discussed LEC with a HCP but without receiving satisfactory answers to their inquiries. Nearly a quarter of respondents (23 %) had not engaged in discussions with a HCP about LEC, despite recognizing the need to do so.

**Fig. 2:**
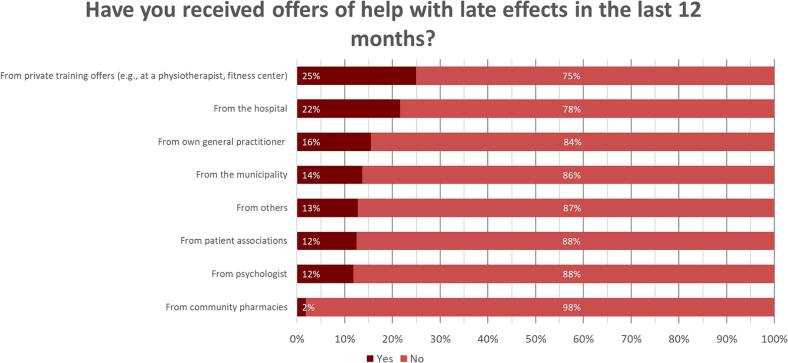
Whether respondents have received help for LEC in the last 12 months and from whom/where.

Fig. 2 illustrates the degree to which respondents received assistance in managing their LEC over the past 12 months and its sources. Twenty-five percent of respondents reported receiving aid from private HCPs such as physiotherapists, personal trainers, and private psychologists. When elaborating the free-text responses on the type of support they received, many respondents mentioned having to proactively seek support themselves, as they were not offered any assistance. Several respondents recounted instances where HCPs expressed a desire to support them but were hindered by financial constraints within the healthcare system. Consequently, several respondents described having to finance the support or treatment they required out of their own pockets.

Many (102) of the free-text responses within the questionnaire reflected experiences with the healthcare system. Among these, 86 entries recounted negative encounters, while 36 highlighted positive experiences. Within the negative experiences, many respondents expressed frustration over inadequate dialogue with HCPs and social workers. Moreover, lack of knowledge and competency among HCPs regarding LEC were recurrent themes in 106 free-text responses. Some respondents also cited inconsistencies in counseling provided by different HCPs as a notable concern. Some respondents would like more time for thorough conversations with their doctors but felt that their doctors were too busy. Furthermore, numerous respondents described that their concerns were not taken seriously by HCPs.

“At the annual check-up at the hospital, I mentioned LEC - as described. The doctor said that if you give it too much attention, it will become a problem.”

Several respondents expressed feeling powerless and alone when navigating the healthcare system. They reported a lack of acknowledgment regarding LEC and the impact on the daily lives of survivors. Furthermore, they described not feeling recognized or heard by HCPs, leaving them with no one to discuss their LEC with:

“There is a lack of recognition of LEC, and it wears you out, as it can be perceived as if the system does not believe that these things really exist and that they are just created in one's mind.”

Many respondents expressed having to initiate discussions regarding LEC with their healthcare providers or even advocating persistently to access the requisite assistance.

Additionally, some respondents articulated a need for greater clarity regarding appropriate contact points for distinct LECs. They also voiced a requirement for assistance navigating available treatment, rehabilitation, and support resources. Furthermore, respondents recounted experiences of encountering different HCPs across appointments, thereby hindering the establishment of trust. This lack of continuity resulted in some respondents feeling passed from one provider to another without a clear focus of responsibility for their care, exacerbating feelings of disconnection and abandonment. Delays in accessing care were also highlighted as a prevalent concern among multiple respondents.

### 3.3 Cancer survivors' use of community pharmacies

Half of respondents had visited a community pharmacy either weekly (6 %) or monthly (44 %) during the past 12 months. Only 3 % of respondents had not visited a pharmacy at least once during the past year.

Respondents were asked about their use of 16 specific types of products available at community pharmacies (see Table 2 – Types of products available at community pharmacy used by cancer). More than half of respondents (53 %) reported having used over-the-counter medicines during the past six months, with an equivalent proportion having used vitamins/minerals.

**Table 2:** – Types of products available at community pharmacy used by cancer survivors.

During the past 6 months, have you used one or more of the following (types) of products?	
	Percentage
Over-the-counter medicines (nasal sprays, painkillers, coughs, medicines for diarrhoea and constipation, acid reflux and heartburn, stomach acid, fungus, eczema, etc.)	53 %
Vitamins/minerals in addition to the contents of a regular multi-vitamin	50 %
Ointments for vulnerable skin	32 %
Herbal remedies and other supplements	29 %
Eye drops for dry eyes	27 %
Vaginal tablets and gels for the care of dry mucous membranes in the vagina	15 %
Compression socks	13 %
Insoles for shoes	12 %
Support bandages for knees, elbows, wrists, loins, etc.	11 %
Other	11 %
No, I have not used any of the above	10 %
Products for stimulating hair growth (scalp spray, hair shampoo, tablets)	10 %
Products for incontinence	9 %
Nutritional supplement drinks	8 %
Non-prescription cannabis products	6 %
Gel and patch to stimulate healing of scars	6 %
Heat patches	1 %
Prescription cannabis products	0 %

Seventy percent of respondents reported using products available at community pharmacies to alleviate their LEC. Most respondents (83 %) reported having sought information prior to product utilization, predominantly from hospital facilities (38 %), general practitioners (27 %), or other informational materials (25 %). Merely 13 % of respondents utilized pharmacies as their primary source of information. Approximately 45 % of respondents expressed a need for counseling regarding the usage of these products.

In the free-text responses, respondents articulated a need for comprehensive information regarding LEC management, encompassing support aids (e.g., support bands, compression aids, walking aids), dietary supplements, exercise, and lifestyle modifications. Furthermore, respondents underscored the necessity for guidance on using over-the-counter medications for LEC relief, including products targeting dry mucous membranes, dietary supplements, and pain management. Additionally, respondents emphasized the importance of in-depth knowledge concerning the potential benefits of supportive aids and supplements and their effective integration into their regimens. Lastly, some respondents desired information regarding potential interactions among different products, preferably by consulting HCPs with a holistic understanding of their medication, diet, exercise routine, and supportive aids utilized for LEC management.

### 3.4 Cancer survivors' opinions on community pharmacies' involvement in the management of LEC

Respondents were queried regarding their willingness to accept free pharmacy-based counseling on LEC, with approximately 48 % expressing acceptance, 32 % undecided, and 20 % declining.

Inquiring about desired changes for an enhanced supportive role of pharmacies in LEC management (see Table 3), indicated that only a quarter of respondents directly opposed pharmacy involvement. Predominantly, respondents advocated for improved awareness among cancer survivors regarding possible pharmacy support (39 %), enhanced infrastructure with discreet counseling areas (33 %), and increased knowledge among pharmacy staff concerning cancer (18 %), and available referral options for LEC support (18 %).

**Table 3:** – reasons for greater involvement of community pharmacies in management of LEC.

What would have to be different if the pharmacy were to play a greater role in alleviating your symptoms/late effects after cancer?	
	Percentage
That I had known / been informed about what the pharmacy could help me with	39 %
That there were better / more discreet conditions to talk to the staff at the pharmacy in	33 %
I do not want the staff at the pharmacy to play a role regarding my late effects	25 %
That the staff at the pharmacy had more knowledge about options for referral for help and support (e.g., for remedying/relieving late effects)	18 %
That the staff at the pharmacy had more knowledge about my cancer and treatment of cancer	18 %
Do not know	15 %
That there was more time to talk to the staff at the pharmacy	12 %
That the staff at the pharmacy had asked about it	10 %
The role of the pharmacy regarding my late effects has been appropriate	6 %
That the staff at the pharmacy had been more understanding and showed interest in me and my cancer	4 %

Some free-text responses concerned the expected role of pharmacies and competencies of pharmacy staff. Some respondents expressed skepticism regarding the adequacy of pharmacy knowledge concerning LEC and cancer in general. A few respondents elaborated in the free-text responses that they perceive pharmacy staff to possess limited knowledge about supplements and supportive aids. Others noted that pharmacy staff may be solely knowledgeable about supplements, lacking proficiency in other areas.

Some respondents were also skeptical regarding the pharmacy's potential to support LEC management. A few concerns were raised regarding the potential conflict of interest, suspecting pharmacies prioritizing profit by promoting products in their counseling. Consequently, a few respondents expressed skepticism towards pharmacy counseling, citing doubts about its trustworthiness:

“The pharmacy is a point of sale, and employees at the pharmacy are salespersons with health-related knowledge. I will never accept advice or guidance from companies that earn money from selling me products.”

Additionally, a few respondents highlighted that they perceive pharmacy staff as unresponsive to their concerns, while others generally lack trust in pharmacy staff. Some respondents attributed their discomfort in discussing personal and private matters to their unfamiliarity or absence of a personal relationship with pharmacy staff:

“I do not want to talk to a random person at the pharmacy. My doctor knows me, and I think these are private topics I do not want to discuss with others.”

Practical conditions within the pharmacy were also highlighted in some responses, echoing the findings in the quantitative data. Several of the free-text responses concerning pharmacies raised concerns about the lack of discretion within the pharmacy environment, citing worries about being overheard by neighbors or acquaintances. Additionally, a few respondents recounted instances where they felt their privacy was compromised while purchasing medicine at the pharmacy:

“Generally, picking up medicine from pharmacies is really not nice. There is a great lack of discretion when counselling on medicine. This often means that I refuse advice, for example, since everyone can hear my statements about my illness.”

Most critical free-text responses were provided in the final field where respondents could comment on the questionnaire. However, a subset of respondents offered constructive suggestions in the open-ended questions throughout the questionnaire. For example, a few respondents proposed establishing private counseling rooms within the pharmacy to enhance community pharmacy involvement. Additionally, one respondent highlighted a lack of awareness about the support community pharmacies can offer for managing LEC, despite the respondent having an excellent pharmacy.

## 4 Discussion

The findings of this study suggest that a majority of cancer survivors experience LEC and express a need for improved LEC management within the healthcare system. Cancer survivors frequently visit community pharmacies and utilize available products to alleviate their LEC symptoms. Moreover, they are receptive to the idea of community pharmacies assuming a counseling role in LEC management. However, they expressed a need for better preparation of both pharmacy organization and staff to effectively fulfill this role.

### 4.1 Cancer survivors' LECs

The study revealed that cancer survivors experienced LEC to a high degree, with fatigue being the most prevalent LEC. International studies on LEC prevalence also emphasize the great extent of LECs. For instance, a German survey conducted by Schmidt et al. (2022), involving 1348 disease-free survivors who had suffered from various cancer types approximately four years post-diagnosis, reported that the most frequently encountered problems with moderate to severe impact included loss of physical performance (36.3 %), fatigue (35.1 %), sexual problems (34.7 %), sleep disturbances (34.1 %), and arthralgia (33.8 %).^42^ In collaboration with the Norwegian Cancer Society, an online cross-sectional study was undertaken among 706 members of their user panel who had experienced present or previous cancer in 2021. The study revealed that a significant majority of respondents (83 %) experienced LEC, primarily manifesting as fatigue (59.2 %), sleep disorders (41.5 %), hot flashes (39.2 %), nerve damage (38.0 %), and pain (36.6 %).^43^

### 4.2 Cancer survivors' information and counseling needs regarding LEC

A significant proportion (75 %) of respondents in our study expressed dissatisfaction with the level of counseling received on LEC. One-third (33 %) reported having discussed LEC with a HCP without receiving satisfactory answers, while nearly a quarter (23 %) had refrained from discussing LEC with a HCP despite feeling the need to do so. These findings corroborate with results from the DCS barometer surveys conducted in 2019 and 2023. The 2019 survey, involving 3153 cancer patients 2.5 years post-diagnosis, revealed that approximately 60 % of respondents expressed a need for support in managing LEC, with nearly half lacking knowledge about symptoms and a third uncertain about whom to contact for assistance.^6^ In the 2023 survey, conducted 4–8 months post-diagnosis, more than half (52 %) of respondents had not discussed with HCPs how their general practitioner could assist them, and 40 % were unaware of support opportunities within their local community.^44^

Similar needs and experiences among cancer survivors are evident in various international studies. Schmidt et al. (2022) highlighted inadequate support, particularly for non-medically threatening or non-treatable issues in Germany,^42^ while a survey by Geller et al. (2014) in Vermont identified unmet needs in emotional, social, spiritual, economic, and legal domains, with common unmet needs including stress reduction and information about LEC.^45^ An American survey by Barg et al. (2007) indicated significant emotional, physical, and financial needs,^46^ while a study by Beckjord et al. (2008) on health-related information needs of survivors found that more than half of respondents desired additional information about expected LEC from cancer treatments.^47^ A systematic review by Hoekstra et al. (2014) across various countries, including the UK, USA, Canada, Italy, and Denmark, identified psychosocial and informational needs among cancer survivors.^48^ The consistent findings across these studies suggest alignment between the experiences and needs of cancer survivors in Denmark and other countries.

### 4.3 Cancer survivors need counseling on products for LEC which pharmacies can provide

In the current study, more than half of respondents used over-the-counter drugs or vitamins/minerals in the past six months. Among them, 70 % utilized these products to alleviate LEC. This aligns with findings from a cross-sectional study by the Norwegian Cancer Society, where 13 % of participants reported using herbal and natural remedies for LEC.^43^

Although these products are available in pharmacies, only 13 % of respondents in the current study actively sought information from pharmacies on their use. Additionally, nearly half of respondents (45 %) expressed a need for counseling regarding product usage. This underscores a low level of engagement with pharmacies among respondents, which may indicate a lack of awareness about the valuable information and guidance pharmacies can provide.

Under Danish pharmacy law, pharmacies are mandated to provide relevant counseling and information on medicines.^49^ Additionally, Good Pharmacy Practice guidelines stipulate that pharmacies should offer counseling on medicines use and other products, as well as engage in health promotion activities.^50^ Recent studies in Denmark have demonstrated active counseling on self-care by pharmacies, including discussions about symptoms and non-prescription products like herbal remedies, suggesting a potential avenue for addressing the needs of cancer survivors seeking relief from LEC through pharmacy services.^13^^,^^51^

In the current study, 97 % of respondents visited the pharmacy annually, aligning closely with the general Danish population, where community pharmacies engage with 94 % of adults annually.^9^ Notably, half visited weekly (6 %) or monthly (44 %), suggesting frequent visits. This underscores the potential for pharmacies to serve as a valuable resource for counseling on products used to alleviate LEC, in accordance with legal requirements and established pharmacy practices.

### 4.4 The cancer survivors are open to the idea of involving community pharmacies in cancer care

In the current study, 25 % of respondents expressed reluctance regarding involving pharmacy staff in LEC management, while 75 % showed varying degrees of openness. Similar positive attitudes towards engaging community pharmacies in cancer care are evident in other studies. A British study on cancer pain patients found that most patients considered pharmacy consultations acceptable, particularly appreciating telephone consultations for those with mobility challenges.^52^ Additionally, a French study reported positive perceptions of community pharmacists among cancer patients, highlighting their willingness to share information and receive advice from pharmacy staff.^53^

As previously noted, various studies have successfully engaged pharmacists in cancer awareness and screening,^16^, ^17^, ^18^, ^19^, ^20^, ^21^, ^22^, ^23^, ^24^, ^25^, ^26^ adherence counseling for treatment side effects,^27^, ^28^, ^29^, ^30^ and cancer prevention and care.^31^^,^^32^ Additionally, a study demonstrated the feasibility of a lifestyle intervention post-prostate cancer treatment in British community pharmacies, showing high acceptability among participants.^33^ A follow up study on patient-reported outcomes from the intervention revealed significant improvements in patient activation, exercise, and diet due to the intervention. Moreover, community pharmacies were effective in addressing LEC post-prostate cancer treatment, such as sexual dysfunction, pain, and discomfort.^31^

Despite some patients in the studies undergoing cancer treatment, the overall positive attitude towards involving community pharmacies in cancer-related interventions and counseling aligns with the findings in the current study. This suggests potential for further research on integrating Danish community pharmacies in management of LEC among cancer survivors.

### 4.5 Perceived role of the community pharmacies for the management of LEC

In the current study, 39 % of cancer survivors lacked awareness of how community pharmacies could assist with LEC. Some perceived pharmacy staff primarily as salespersons, raising concerns about the credibility of pharmacy counseling due to perceived profit motives. Previous research similarly indicates that cancer survivors often view pharmacies solely as places to collect medication.^54^, ^55^, ^56^ This misalignment may be further compounded by the fact that pharmacies do not dispense oncology medications, which might have contributed to the lack of association between pharmacies and cancer-related care among cancer survivors. A recent Danish study by Buhl et al. (2023) discovered that community pharmacy staff also perceived public perceptions of the pharmacy's role as a barrier to counseling cancer survivors.^57^ Improved communication and public education regarding the role and capabilities of community pharmacies are crucial to bolstering the credibility of these establishments in supporting cancer survivors with LEC.

### 4.6 Pharmacies and staff need upgrading to accommodate cancer survivors

To enhance community pharmacy involvement in the management of LEC, cancer survivors in the current study stressed the importance of improved discretion and knowledge among pharmacy staff regarding cancer, LEC, and potential treatment options such as nutritional supplements. Additionally, some respondents expressed discomfort in discussing personal topics due to unfamiliarity with pharmacy staff.

According to Buhl et al. (2023), primary barriers to counseling cancer survivors at community pharmacies, as perceived by pharmacy staff, include a lack of knowledge and training materials on cancer and LEC, as well as the absence of guidelines for communicating with cancer patients. Pharmacy staff have shown interest in receiving more education, particularly regarding LEC treatment and communication with cancer patients.^57^

Discretion concerns in community pharmacies extend beyond cancer diagnoses. A 2021 systematic review identified that a lack of privacy in community pharmacies hinders patient engagement in medication counseling with pharmacists.^58^ In an Australian study involving mental health consumers and carers, respondents emphasized the need for greater discretion in various aspects, such as patient name calls and medication pack handling.^59^^,^^60^ Similarly, participants in a 2010 UK study on cardiovascular screening cited privacy and confidentiality as barriers to participation.^61^ In a 2024 cross-sectional study from Jordan, nearly half (45,7 %) of participants from the public perceived a lack of privacy in community pharmacies.^62^,^63^ Additionally, a 2023 study from Ireland revealed that pharmacists feel constrained in their ability to exercise professional discretion in providing optimal care to patients due to current pharmacy regulations.^64^

In summary, ensuring discretion, addressing staff training gaps, and providing communication guidelines for interacting with cancer patients are essential considerations for involving community pharmacies in counseling LEC survivors.

### 4.7 Limitations

One limitation of this study is the potential for some of the adverse events reported by patients to be unrelated to their cancer treatment and therefore not classified as LEC. It is possible that certain symptoms may have been caused by the use of other medications or underlying health conditions unrelated to cancer. This limitation highlights the complexity of accurately attributing adverse events to cancer treatment alone.

The questionnaire employed in this study demonstrated some degree of validity, since it utilized previously validated instruments tailored to the target population, language, and culture, including those specifically designed for health purposes and cancer survivors.^6^^,^^36^, ^37^, ^38^ Minimal modifications were made to the questionnaire, with adapted questions undergoing testing within the target population and new questions being piloted to enhance reliability. Furthermore, the validity of the questionnaire was bolstered by the consistency of results regarding LEC compared to similar studies conducted in other countries, indicating content validity.^42^^,^^45^, ^46^, ^47^, ^48^

Although a response rate of 58 % suggests a willingness among User Panel members to participate in the survey, the 42 % non-response rate introduces potential selection bias. Additionally, the demographic composition of the sample, while representative of cancer survivors from all regions of Denmark, displayed an overrepresentation of women and individuals with higher education, possibly limiting its generalizability to the broader Danish cancer population. Nonetheless, including a substantial percentage (47 %) of cancer-free individuals relevant for future counseling efforts on LEC in community pharmacies adds value to the study. Despite potential sampling bias, the national representation of the User Panel from all regions of Denmark ensures geographic diversity, thereby strengthening results.

While qualitative results may not be inherently generalizable, the study's findings on needs and attitudes, derived from quantitative and qualitative responses, align with conclusions from similar studies in other countries. A notable strength of this study is the integration of qualitative and quantitative results.

### 4.8 Perspectives

Several crucial areas warrant further investigation to optimize the involvement of community pharmacies in supporting cancer survivors with LEC. One such area involves identifying support needs from the perspective of cancer survivors. Understanding what cancer survivors perceive community pharmacies can assist with in LEC management is essential for developing relevant pharmacy services and interventions that meet their expectations and needs.

Additionally, community pharmacy staff must possess the necessary knowledge and competencies to support LEC management effectively. This necessitates comprehensive training on LEC, counseling techniques, and communication skills tailored to the specific needs of cancer survivors. Further studies should explore the content of such training programs.

To address the limitation that some adverse events may be unrelated to cancer treatment, future studies should analyze patients' full medication profiles. This will help distinguish between true LEC and other events, and identify needs for medication review and therapy management, ultimately improving patient care.

By addressing these critical aspects the way can be paved for community pharmacies to assume a more significant role in supporting cancer survivors with LEC, thereby enhancing their quality of life and overall well-being.

Several international studies have highlighted a positive attitude towards integrating community pharmacies in cancer-related interventions and counseling, aligning with the sentiments observed in this study. As such, the findings of this research may have broad applicability to similar cancer survivor populations worldwide.

While community pharmacies offer a promising avenue for support, efforts to enhance and communication skills and public awareness of the pharmacy's potential role are crucial to maximizing their effectiveness in supporting cancer survivors.

## 5 Conclusion

This study underscores the significant challenges faced by cancer survivors when trying to manage LEC and the potential role of community pharmacies in addressing these challenges. The findings reveal the prevalent experience of LEC among cancer survivors and the lack of adequate counseling on symptom management.

Cancer survivors demonstrate a willingness to engage community pharmacies in LEC management, yet concerns persist regarding pharmacies' perceived role and discretion issues. Thus, there is a clear need to enhance pharmacy staff knowledge on cancer care and upgrade pharmacy facilities to better accommodate cancer survivors' counseling needs.

## Funding

This work was funded through the project “Knowledge about Quality in the Cancer Patient Course (VOK2)” based at The Danish Cancer Society.

## CRediT authorship contribution statement

**Nadia Lund Olsen:** Writing – review & editing, Writing – original draft, Visualization, Validation, Project administration, Methodology, Investigation, Formal analysis, Data curation, Conceptualization. **Ramune Jacobsen:** Writing – review & editing, Writing – original draft, Validation, Supervision, Methodology, Conceptualization. **Linda Aagaard Thomsen:** Writing – review & editing, Writing – original draft, Validation, Supervision, Methodology, Investigation, Funding acquisition, Data curation, Conceptualization. **Lotte Stig Nørgaard:** Writing – review & editing, Writing – original draft, Validation, Supervision, Project administration, Methodology, Formal analysis, Conceptualization.

## Declaration of competing interest

The authors declare that they have no known competing financial interests or personal relationships that could have appeared to influence the work reported in this paper.

## Appendix A Supplementary data

Table of questions from the questionnaire sent to the DCS User Panel Image 1
