# Supplementary material for: Involving community pharmacies in management of late effects of cancer treatment: Opinions from cancer survivors
Source: Explor Res Clin Soc Pharm. 2024 Sep 21;16:100514. doi: 10.1016/j.rcsop.2024.100514 (PMC11470256; doi:10.1016/j.rcsop.2024.100514)
Supplement: Supplementary file 1 — Table of questions from the questionnaire sent to the DCS User Panel [file mmc1.docx]

# Supplementary material

The questionnaire

Table 4 table of questions from questionnaire sent to the DCS User Panel

| **Qno.** | **Question** | **Subquestion** | **Possible Answers** |
| --- | --- | --- | --- |
| Q1 | What is the status of your cancer treatment? You can choose multiple answers | | I am receiving treatment at the hospital aimed at eliminating the cancer (e.g., chemotherapy, radiation therapy, surgery) |
|  |  |  | I no longer have cancer, but I am receiving treatment at the hospital/with a general practitioner to prevent the cancer from returning |
|  |  |  | I am receiving treatment at the hospital/with a general practitioner to prevent the cancer from progressing further |
|  |  |  | I am receiving palliative care at the hospital/with a general practitioner, as the cancer cannot be cured |
|  |  |  | I am no longer receiving any cancer treatment at the hospital because the cancer is gone |
|  |  |  | I have never received treatment for my cancer |
| Q2 | If you have received treatment, how long has it been since you completed it? | | The treatment is not completed |
|  |  |  | 0-1 year |
|  |  |  | 2-5 years |
|  |  |  | 6-10 years |
|  |  |  | 11-15 years |
|  |  |  | 16-20 years |
|  |  |  | 21 years or longer |
| Q3 | During or after your cancer/ treatment, have you experienced any of the following issues/problems? Choose one option per line | Anxiety | Not at all - A little - Some - A lot |
|  |  | Depression | Not at all - A little - Some - A lot |
|  |  | Troublesome fatigue | Not at all - A little - Some - A lot |
|  |  | Lymphedema (swollen arms/legs) | Not at all - A little - Some - A lot |
|  |  | Less desire for sexual activity than before the diagnosis | Not at all - A little - Some - A lot |
|  |  | Dry vaginal mucous membranes | Not at all - A little - Some - A lot |
|  |  | Incontinence (unintentional urination) | Not at all - A little - Some - A lot |
|  |  | Tingling or loss of sensation in fingers or toes | Not at all - A little - Some - A lot |
|  |  | Changed taste or sense of smell | Not at all - A little - Some - A lot |
|  |  | Dry mouth | Not at all - A little - Some - A lot |
|  |  | Difficulty swallowing | Not at all - A little - Some - A lot |
|  |  | Weight loss | Not at all - A little - Some - A lot |
|  |  | Weight gain | Not at all - A little - Some - A lot |
| Q4 | Have you experienced any other issues/problems besides those listed above? | | Yes, please describe: _________ |
|  |  |  | No |
| Q5 | Have you missed any form of counseling, help, or other support related to your issues/problems? | | Yes, to a large extent |
|  |  |  | Yes, to some extent |
|  |  |  | Yes, but only a little |
|  |  |  | No, not at all |
|  |  |  | Do not know/not relevant |
| Q6 | Please indicate which problems you missed counseling or help with: You can choose multiple answers | | Anxiety |
|  |  |  | Depression |
|  |  |  | Troublesome fatigue |
|  |  |  | Lymphedema (swollen arms/legs) |
|  |  |  | Less desire for sexual activity than before the diagnosis |
|  |  |  | Dry vaginal mucous membranes |
|  |  |  | Incontinence (unintentional urination) |
|  |  |  | Tingling or loss of sensation in fingers or toes |
|  |  |  | Changed taste or sense of smell |
|  |  |  | Dry mouth |
|  |  |  | Difficulty swallowing |
|  |  |  | Weight loss |
|  |  |  | Weight gain |
|  |  |  | If other, please specify |
| Q7 | Have you talked to a healthcare professional about your late effects after cancer? | | Yes, and we had a good conversation |
|  |  |  | Yes, but I felt my questions were not answered |
|  |  |  | No, but I would have liked to talk to someone |
|  |  |  | No, I have not felt the need |
| Q8 | In the past 12 months, have you accepted offers of help for late effects? By 'offers' we mean e.g., rehabilitation, targeted courses, professional guidance, or personal counseling. Choose one option per line | From the municipality | Yes - No |
|  |  | From your general practitioner | Yes - No |
|  |  | From the hospital | Yes - No |
|  |  | From the pharmacy | Yes - No |
|  |  | From patient associations | Yes - No |
|  |  | From psychologists | Yes - No |
|  |  | From private training offers (e.g., at a physiotherapist, fitness center) | Yes - No |
|  |  | From others | Yes - No |
| Q9 | Have you talked to the pharmacy staff about late effects after cancer? | | Yes |
|  |  |  | No |
| Q10 | What did you talk to the pharmacy staff about? You can choose multiple answers | | About physical problems (e.g., dry mouth, pain, incontinence, diarrhea, or constipation) |
|  |  |  | About psychological problems (e.g., anxiety, sadness, or worries) |
|  |  |  | About what I can do myself to feel better |
|  |  |  | Other, please specify |
| Q11 | Did you or the pharmacy staff bring up the topic? | | I brought up the topic myself |
|  |  |  | The pharmacy staff asked about it |
| Q12 | In the past 6 months, have you used one or more of the following types of products? You can choose multiple answers | | Vitamins/minerals beyond what is in a regular vitamin pill |
|  |  |  | Herbal medicines and other dietary supplements |
|  |  |  | Eye drops for dry eyes |
|  |  |  | Products to stimulate hair growth (scalp spray, shampoo, tablets) |
|  |  |  | Creams for vulnerable skin |
|  |  |  | Gel and plasters to stimulate scar healing |
|  |  |  | Suppositories and gels for the care of dry vaginal mucous membranes |
|  |  |  | Support stockings |
|  |  |  | Support bandages for knees, elbows, wrists, lower back, etc. |
|  |  |  | Insoles for shoes |
|  |  |  | Products for incontinence |
|  |  |  | Heat patches |
|  |  |  | Over-the-counter medicines (nasal spray, pain relievers, cough suppressants, remedies for diarrhea and constipation, remedies for acid reflux and heartburn, remedies for excessive stomach acid, antifungal medications, eczema medications, etc.) |
|  |  |  | Nutritional drinks |
|  |  |  | Cannabis products without a prescription |
|  |  |  | Cannabis products with a prescription |
|  |  |  | Other, please specify: |
|  |  |  | No, I have not used any of the above |
| Q13 | What is the reason you have used the above products in the past 6 months? You can choose multiple answers | | To alleviate or relieve issues/late effects |
|  |  |  | To improve my physical well-being |
|  |  |  | To improve my mental well-being |
|  |  |  | To prevent the cancer from returning |
|  |  |  | Because I was encouraged/recommended to try it |
|  |  |  | Other |
| Q14 | Did you seek information about the products before you started using them? You can choose multiple answers | | Yes, from one or more healthcare professionals at the hospital |
|  |  |  | Yes, from a general practitioner |
|  |  |  | Yes, from the pharmacy |
|  |  |  | Yes, from others who have used alternative treatments (e.g., in Facebook groups) |
|  |  |  | Yes, in information materials (e.g., brochures, leaflets, or on the internet) |
|  |  |  | Yes, from family, friends, or acquaintances |
|  |  |  | No, I did not seek information |
| Q15 | Have you missed any form of counseling, help, or other support regarding these products? | | Yes, to a large extent |
|  |  |  | Yes, to some extent |
|  |  |  | Yes, but only a little |
|  |  |  | No, not at all |
|  |  |  | Do not know/not relevant |
| Q16 | How often have you visited a pharmacy in the last 12 months? | | Almost daily or daily |
|  |  |  | Weekly |
|  |  |  | Monthly |
|  |  |  | 5-10 times |
|  |  |  | 2-4 times |
|  |  |  | Once |
|  |  |  | I have not visited a pharmacy in the past 12 months |
| Q17 | What should be different if the pharmacy were to play a larger role in alleviating your issues/late effects after cancer? You can choose multiple answers | | That I had known/been informed about what the pharmacy could help me with |
|  |  |  | That the pharmacy staff had asked about it themselves |
|  |  |  | That the pharmacy staff had more knowledge about my cancer and treatment |
|  |  |  | That there was more time to talk to the pharmacy staff |
|  |  |  | That there were better/more discreet conditions to talk to the pharmacy staff in |
|  |  |  | That the pharmacy staff had more knowledge about referral options for help and support (e.g., for alleviating/relieving late effects) |
|  |  |  | That the pharmacy staff had been more understanding and showed interest in me and my cancer |
|  |  |  | The pharmacy's role regarding my late effects has been adequate |
|  |  |  | I do not want the pharmacy staff to play a role regarding my late effects |
|  |  |  | Do not know |
| Q18 | If you were made aware of a free counseling offer on late effects at your local pharmacy, would you choose to participate in such an offer? | | Yes |
|  |  |  | No |
|  |  |  | Do not know |
| Q19 | Do you live with others? Choose one option per line | I live with a spouse/partner | Yes - No |
|  |  | I live with a child/children under 16 years | Yes - No |
|  |  | I live with others aged 16 years or older | Yes – No |
